# What is the current status of primary care in the diagnosis and treatment of patients with vertigo and dizziness in Switzerland? A national survey

**DOI:** 10.3389/fneur.2023.1254080

**Published:** 2023-09-07

**Authors:** Andreas Zwergal, Georgios Mantokoudis, Dierik Heg, Hassen Kerkeni, Suzie Diener, Roger Kalla, Athanasia Korda, Claudia Candreia, Antje Welge-Lüssen, Alexander A. Tarnutzer

**Affiliations:** ^1^German Center for Vertigo and Balance Disorders (DSGZ), Ludwig Maximilian University Hospital, Munich, Germany; ^2^Department of Neurology, Ludwig Maximilian University Hospital, Munich, Germany; ^3^Department of Otorhinolaryngology, Head and Neck Surgery, Lnselspital, Bern University Hospital, University of Bern, Bern, Switzerland; ^4^Clinical Trial Unit Bern, University of Bern, Bern, Switzerland; ^5^Department of Neurology, Lnselspital, Bern University Hospital, University of Bern, Bern, Switzerland; ^6^Practice Neurology St. Gallen, St. Galen, Switzerland; ^7^Department of Otorhinolaryngology, Head and Neck Surgery, Cantonal Hospital Lucerne, Lucerne, Switzerland; ^8^Department of Otorhinolaryngology, Head and Neck Surgery, University Hospital Basel, Basel, Switzerland; ^9^Neurology, Cantonal Hospital of Baden, Baden, Switzerland; ^10^Faculty of Medicine, University of Zurich, Zurich, Switzerland

**Keywords:** vertigo, dizziness, survey, bedside examination, primary care, diagnosis

## Abstract

**Background:**

Vertigo and dizziness are among the most frequent presenting symptoms in the primary care physicians' (PCPs) office. With patients facing difficulties in describing their complaints and clinical findings often being subtle and transient, the diagnostic workup of the dizzy patient remains challenging. We aimed to gain more insights into the current state of practice in order to identify the limitations and needs of the PCPs and define strategies to continuously improve their knowledge in the care of the dizzy patient.

**Materials and methods:**

Board-certified PCPs working in Switzerland were invited to participate in an online survey. A descriptive statistical analysis was performed, and prospectively defined hypotheses were assessed using regression analyses.

**Results:**

A vast majority of participating PCPs (*n* = 152) were familiar with the key questions when taking the dizzy patient's history and with performing provocation/repositioning maneuvers when posterior-canal benign paroxysmal positional vertigo (BPPV) was suspected (91%). In contrast, strong agreement that performing the alternating cover test (21%), looking for a spontaneous nystagmus with fixation removed (42%), and performing the head-impulse test (47%) were important was considerably lower, and only 19% of PCPs were familiar with lateral-canal BPPV treatment. No specific diagnosis could be reached in substantial fractions of patients with acute (35% [25; 50%], median [inter-quartile range]) and episodic/chronic (50% [40; 65.8%]) dizziness/vertigo. Referral to specialists was higher in patients with episodic/chronic dizziness than in acutely dizzy patients (50% [20.3; 75] vs. 30% [20; 50]), with younger PCPs (aged 30–40 years) demonstrating significantly increased odds of referral to specialists (odds ratio = 2.20 [1.01–4.81], *p* = 0.048).

**Conclusion:**

The assessment of dizzy patients takes longer than that of average patients in most primary care practices. Many dizzy patients remain undiagnosed even after a thorough examination, highlighting the challenges faced by PCPs and potentially leading to frequent referrals to specialists. To address this, it is crucial to promote state-of-the-art neuro-otological examination and treatment techniques that are currently neglected by most PCPs, such as “HINTS” and lateral-canal BPPV treatment. This can help reduce referral rates allowing more targeted treatment and referrals.

## Introduction

Vertigo or dizziness (1) are among the most frequently reported reasons for seeking medical advice. The 1-year prevalence for dizziness in national surveys was found to be 14.8–35.6% (2–5). The prevalence of dizziness increased with age (6), reaching 45% for adults aged 65 years or above (7) and 54% at 79 years of age (8). The impact of vertigo and dizziness on the patient's quality of life is significant, with an interruption of daily activities in 67–80% of patients (6, 9), sick leave, or medical consultations in 80% of patients (9). Bronstein and colleagues reported that 27% change jobs, 21% give up work, and 50% note reduced efficacy at work (10). Furthermore, an increase of 12 times in the odds of falling with 10% major injuries was observed (11). Vertigo and dizziness contribute the most to the burden of disability among all healthcare problems in a population cohort older than 80 years (12).

Due to the high lifetime prevalence of dizziness (17–30%) and vertigo (3–10%) (13), approximately 40% of all Americans will therefore seek medical attention at some point in their lives (14). In a large National Ambulatory Care Survey in the United States (20.6 million adult visits for dizziness from 2013 to 2015), the prevalence rate was 8.8/1,000 visits (15). Notably, the majority of visits for dizziness were to primary care physicians (PCPs, 51.9%), whereas specialists such as otorhinolaryngologists (13.3%) and neurologists (9.6%) were involved less frequently (15). In a systematic review, the consultation prevalence of vertigo/dizziness in primary care practice varied between 1.0 and 15.5% (16). In a Dutch survey, the 1-year prevalence of dizziness in family practice in patients aged 65 years or older was 8.3% (17). In primary care practice, the fraction of patients presenting with a leading symptom of vertigo or dizziness receiving no specific diagnosis varies significantly between studies (range 0.0–80.2%) (16). After 1 year of follow-up, family doctors were still unable to specify a diagnosis in 39% of dizzy patients (17), reporting a symptom diagnosis (e.g., vertigo/dizziness, fainting/syncope, or general weakness/tiredness) only. In another study, patients received a medical diagnosis of “unspecified dizziness” in 75.2% of consultations (15). Surprisingly, only 14.9% of all patients were referred to another physician for further evaluation in this study (15). In another study, only 22% of patients seen by a PCP in the US veterans' health service were referred to specialists (18). Furthermore, physical therapy referral was the exception in peripheral and central vestibular disorders (0.5%) (18), despite its currently known efficacy, for example, unilateral or bilateral vestibulopathy (19).

Considering the reported difficulties in identifying specific diagnoses and the low rate of referral to specialists in various countries around the world, this uncovers significant limitations in the care of the dizzy patient. At the same time, most of these epidemiological studies are based on surveys performed more than 10–20 years ago and thus may not reflect the implementation of diagnostic and therapeutic approaches promoted more recently such as applying the HINTS [Head-Impulse, Nystagmus, and Test of Skew (20)] in the acutely dizzy patient. In addition, previous studies may not be applicable to the Swiss healthcare system. We, therefore, investigated the current state of care for the dizzy patient in the highly developed Swiss healthcare system and how the diagnostic workup of the dizzy patient could be improved. The primary aim of this study was therefore to (1) gain more knowledge about the current exposure of both PCPs and specialists to dizzy patients, (2) identify limitations and pitfalls in the diagnostic workup and in the interaction between different specialties (generalists and specialists), and (3) ask for specific needs of the involved specialties. To achieve these aims, online surveys were designed for both PCPs and specialists. In this publication, we report on the current status of care from the perspective of the PCPs, whereas unmet needs, potential educational approaches, and the specialists' perspective are addressed in companion papers.

## Materials and methods

### Design of the questionnaire

For this survey-based study, a structured anonymous online questionnaire was designed by the authors (AZ, GM, and AAT), targeting board-certified PCPs (entitled “general internal medicine”) working in private practice in Switzerland (see Appendix for the full questionnaire). Three main sections were defined to address the pre-specified key aims of the study. While the first section focused on the current situation in the assessment of the dizzy patient by PCPs, the second section addressed limitations faced by the PCPs in the diagnostic workup and in the treatment of the dizzy patient. In the third section, potential strategies to improve the standard of care of the dizzy patient and the interaction between generalists and specialists were discussed, and the value of different teaching formats was evaluated. At the very beginning of the questionnaire, key epidemiological information was collected including the setting of the PCPs' office (location, number of physicians employed), years of professional experience, and professional background.

The estimated time needed to fill out the questionnaire was 20–25 min. The questionnaire was available in both German and French languages, and the translation from German to French was supervised by a native French-speaking expert in the field.

### Delivery of the questionnaire and identification of suitable participants

For this online-only questionnaire, we used Survey Monkey (Momentive Global Inc., San Mateo, CA, USA) both for the delivery of the questionnaire to suitable PCPs and for a descriptive analysis of the results of the survey. The survey was open to all board-certified PCPs working in private practice in Switzerland and was sent to suitable physicians based on a database of interested PCPs run by healthbook.ch. In total, 5,668 PCPs were contacted. According to the most recent report of the Swiss Medical Association (FMH), 8,511 PCPs are currently practicing in Switzerland (21). The target sample size was 150 completed surveys, and we aimed for a proportional representation of participants from all parts of Switzerland. Following the distribution of languages spoken in Switzerland, we aimed for 100 questionnaires from PCPs living/working in the German-speaking part of Switzerland and 50 questionnaires from PCPs located in the French or Italian-speaking part of Switzerland (summarized as the “Latin part of Switzerland”). Reimbursement for the completion of the questionnaire to reflect the amount of time and effort spent was provided to each participant. Calls for participation were sent out five times in total to PCPs in the period from January 2022 to February 2022.

### Statistical analysis of the questionnaire

First, a descriptive statistical analysis of the questionnaire was performed, focusing on epidemiological aspects including office size, location, number of dizzy patients seen, diagnostic tests performed, and treatments initiated. Second, univariate and multivariate statistical analyses were run to validate the pre-specified hypotheses. If the *p*-value was smaller than 0.2 in the univariable analysis, then this variable was also included in the multivariable analysis. Statistical support was provided by the clinical trial unit of the University of Bern (Switzerland).

A series of scores to reflect key aspects of the diagnostic workup (both history taking and bedside testing) were predefined by the authors (AZ, GM, and AAT) and were used to correlate with several epidemiological aspects including years of professional experience, location of PCPs' office, and the reported number of dizzy patients evaluated. These scores were graded based on the extent to which the PCPs agreed with a given procedure or the indicated importance of a proposed measure, ranging from 3 points (very important/fully agreed) and 2 points (rather important/partially agreed) to 1 point (rather unimportant/partially disagree) and 0 point (not important at all/disagree at all). All statistical analyses were performed using Stata version 17. Scores were summed and then indexed to 0–100%. Fractional regressions (odds ratios with 95% confidence intervals) are reported for indexed scores; binary dependent variables were analyzed with logistic regressions (odds ratios with 95% confidence intervals). Descriptive statistics report means with standard deviations (±SD), medians with interquartiles (25–75%), counts with percentages (% of non-missing cases), and sample sizes (number of respondents). See **Figure 3** and its legend for a full explanation of each of the scores derived from the respondents' questionnaire items.

## Results

### Epidemiological key aspects of participating PCPs

A total of 152 completed surveys were included, reflecting a response rate of 2.68%. Only a minority of participants (26%) were women. Notably, the age of 62% of participating PCPs was 51 years or older, and that of only 4% was 40 years or younger (for details, see Table 1). PCPs' offices were mainly located in cities (52%) or agglomerations (29%), whereas rural offices were less frequent (19%). The majority of participating PCPs worked alone (36%) or in small offices (2–4 physicians, 39%), and the average (±1 SD) number of years of working experience of participating PCPs was 26.1 ± 8.9 years (minimum = 6 years, maximum = 40 years). On average (±1 SD), participating PCPs saw 23.2 ± 9.3 patients per day, spending 20.7 ± 5.7 min per patient. On a monthly basis, the number of patients seen with a leading symptom of vertigo or dizziness averaged out at 13.0 (±11.3, 1 SD, range: 1–90 patients). In all, 71% of participating PCPs indicated that they spend more time on average with a patient presenting with dizziness or vertigo than with a patient reporting other major complaints.

**Table 1:** Epidemiological key results.

	***n* (%)**
Gender	
Females	40 (26%)
Males	112 (74%)
Age distribution (years)	
30–40	6 (4%)
41–50	52 (34%)
51–60	55 (36%)
>60	39 (26%)
Specialty of participating board-certified physicians^*^	
General internal medicine	151
Surgery	1
Gastroenterology	1
Hematology	1
Cardiology	1
Pediatrics	1
Pneumology	1
Geographical location of PCPs office	
German part of Switzerland	101 (66%)
Latin (i.e., French/Italian speaking) part of Switzerland	51 (34%)
Location of PCPs office—proximity to a city	
On the countryside	29 (19%)
In the agglomeration	44 (29%)
In the city	79 (52%)
Number of physicians working the PCPs office	
1	54 (36%)
2–4	59 (39%)
5–8	17 (11%)
>8	22 (14%)
Years of professional experience (after finishing their studies)	26.1 ± 8.9 years (*n =* 152)
Number of patients seen per day (average ± 1 SD)	23.2 ± 9.3 (*n =* 152)
Time spent per consultation (min, average ± 1 SD)	20.7 ± 5.7 (*n =* 152)
Number of patients seen with a leading symptom of vertigo or dizziness per month (average ± 1 SD)	13.0 ± 11.3 (*n =* 152)
Time spent per consultation for patients presenting with vertigo or dizziness	
As much time as for other patients on average	44 (29%)
More time as for the average patient	108 (71%)

^*^Five participating physicians indicated more than one specialty.

### History taking in patients presenting with vertigo or dizziness

**Figure 1:**
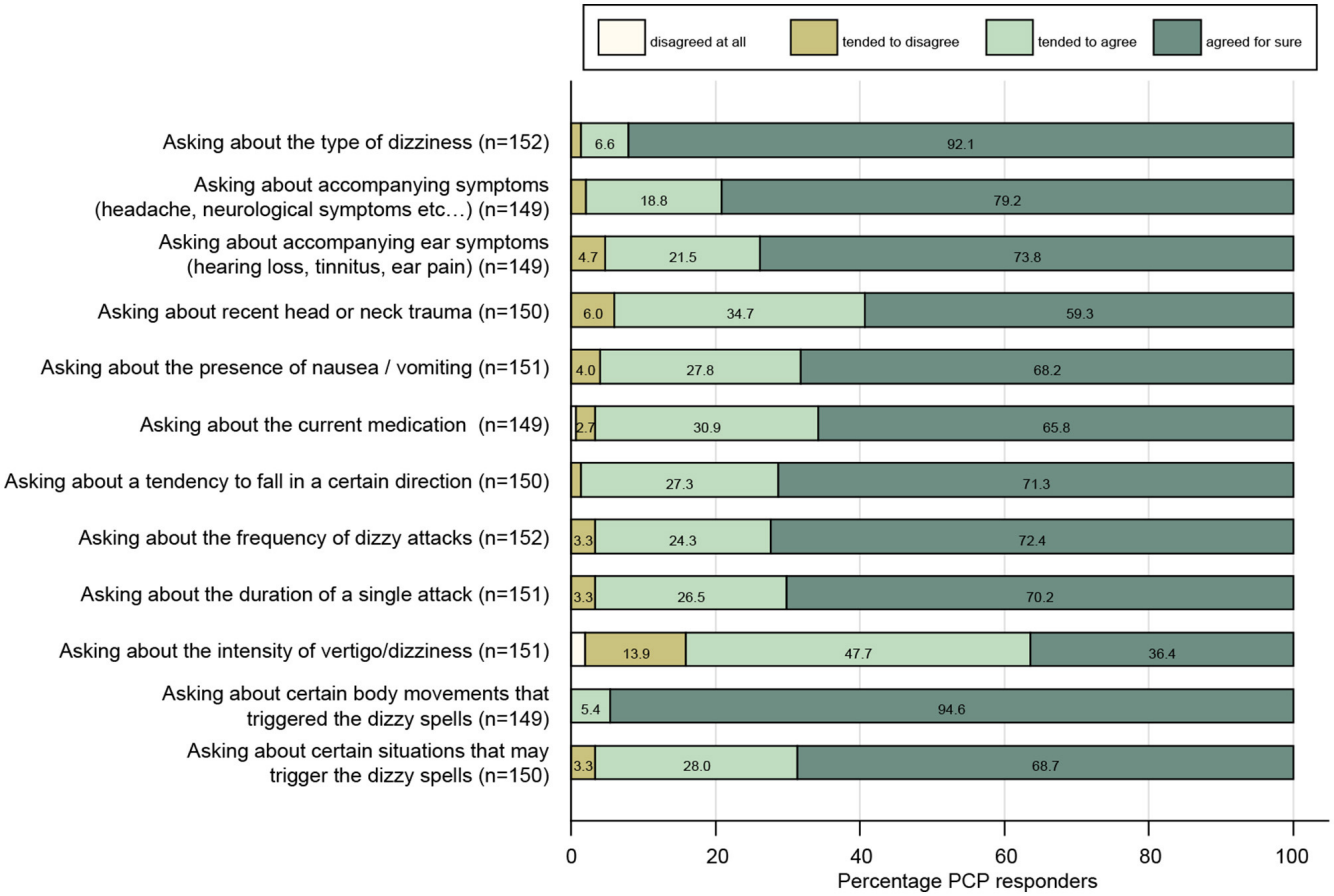
Response patterns of participating PCPs are shown for a series of questions when taking the dizzy patient's history. For each question, the percentage of PCPs and the level of agreement they indicated (ranging from “disagree at all” to “agreed for sure”) are illustrated. For each question, the number (n) of valid replies is provided in brackets.

When taking the dizzy patient's history, participating PCPs agreed for sure or tended to agree to all proposed questions with rates of 83–99% (see Figure 1). The highest rates of strong agreement were found for the questions asking about body movements that triggered dizzy spells (95%), the type of dizziness (92%), and accompanying symptoms (79%), whereas the fractions for strong agreement were lowest for asking about the intensity of vertigo/dizziness (36%), a recent head or neck trauma (59%), and current medication (66%).

### Clinical examination of the patient presenting with vertigo or dizziness

#### Bedside tests performed

**Figure 2:**
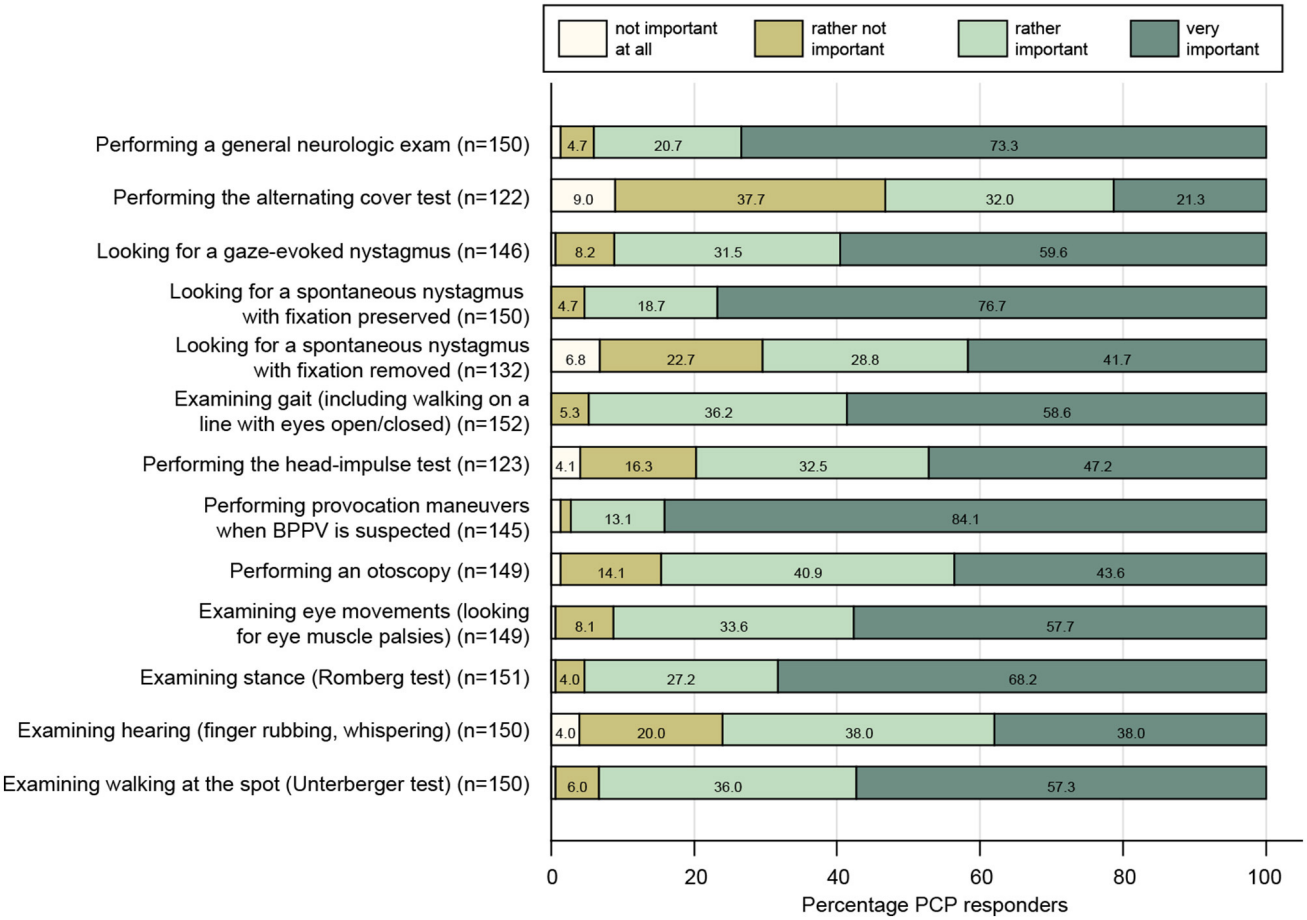
Response patterns of participating PCPs are shown for a series of clinical exams when assessing the dizzy patient. For each question, the percentage of PCPs and the level of importance they indicated (ranging from “not important at all” to “very important”) are illustrated. For each question, the number (n) of valid replies is provided in brackets.

**Figure 3:**
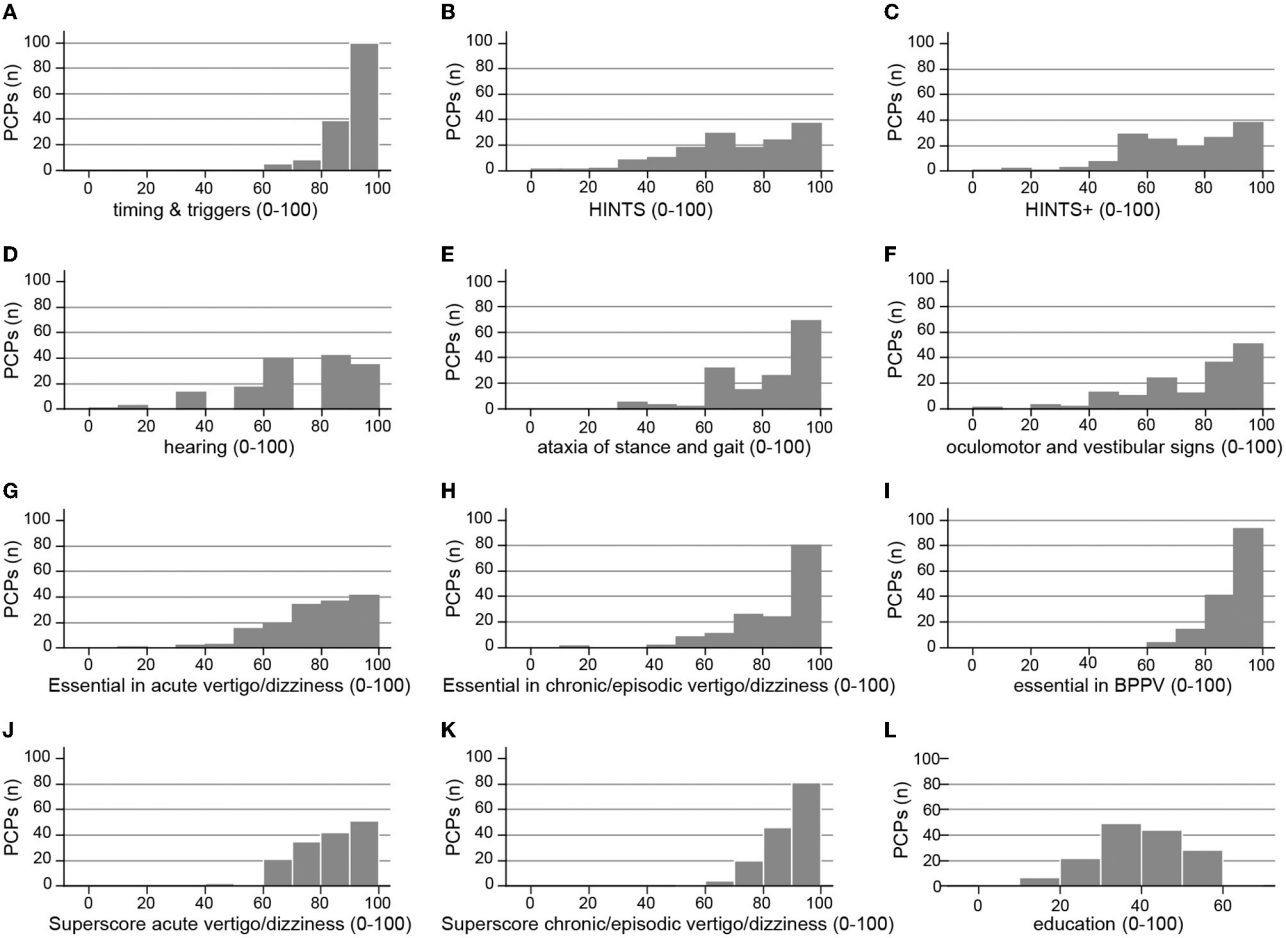
PCPs' performance for various scores is illustrated. This included the following scores: *timing and triggers* **(A)** asking for the frequency and duration of dizzy spells, triggers (specific body movements/positions, specific situations), accompanying symptoms (23), *HINTS* **(B)** performing the head-impulse test, looking for gaze-evoked nystagmus and for skew deviation, *HINTS*+ **(C)** HINTS plus looking for new-onset unilateral hearing loss, *hearing* **(D)** testing for new-onset hearing loss, performing otoscopy, *ataxia of stance and gait* **(E)** assessment of walking on the line (with/without viewing), Romberg test, Unterberger stepping test, *subtle oculomotor and vestibular signs* **(F)** performing HINTS and testing for spontaneous nystagmus with both fixation preserved and removed, “*essential” in acute vertigo/dizziness* **(G)** testing for HINTS+, assessment of walking on the line (with/without viewing), Romberg test and for spontaneous nystagmus with both fixation preserved and removed, “*essential” in episodic/chronic vertigo/dizziness* **(H)** performing provocation maneuvers, the head-impulse test, assessments of walking on the line (with/without viewing), and the Romberg test, “*essential” in suspected BPPV* **(I)** asking for timing and triggers and performing provocation maneuvers, *superscore acute vertigo/dizziness* **(J)** essential in acute vertigo/dizziness and timing and triggers, *superscore for episodic/chronic vertigo/dizziness* **(K)** essential in episodic/chronic vertigo/dizziness and timing and triggers, *education* **(L)** analog media (hands-on courses, workshops, national recommendations, and practical recommendations), and digital media (smartphone apps and webinars).

When examining the dizzy patient at the bedside, PCPs agreed for sure or tended to agree to all proposed tests with rates of 43–95% (see Figure 2). The highest rates of strong agreement that a given bedside test is important were obtained when performing provocation maneuvers when benign paroxysmal positional vertigo (BPPV) is suspected (84%), looking for spontaneous nystagmus with fixation preserved (77%), and performing a general neurological examination (73%). The fraction for strong agreement was lowest for performing the alternating cover test (21%), looking for spontaneous nystagmus with fixation removed (42%), and performing the head-impulse test (47%). A series of scores were calculated to assess the PCP's familiarity with structured history taking in the dizzy patient and with various bedside tests (see Figure 3 and its legend for details). While for some scores most PCPs scored high, such as timing and triggers (Figure 3A), “essential” in BPPV (Figure 3I), and “superscore” in episodic/chronic vertigo or dizziness (Figure 3K), they scored lower in other scores including HINTS(+) (Figures 3B,C), subtle oculomotor and vestibular signs (Figure 3F), and “essential” in acute vertigo/dizziness (Figure 3G). The most variable scores were reached for hearing, oculomotor, and vestibular signs.

When assessing the use of the HINTS [i.e., performing the head-impulse test, testing for gaze-evoked nystagmus and for skew deviation (20)] or its extension [the HINTS+, including new-onset unilateral hearing loss (22)] in patients with acute prolonged dizziness/vertigo, there was no significant correlation with the PCP's age and HINTS (*p* = 0.44) or the HINTS+ (*p* = 0.87). This was also true when adding testing for spontaneous nystagmus (with and without fixation) to the HINTS bedside exam, with no correlation of this “subtle oculomotor and vestibular signs” score with PCPs' age (*p* = 0.56).

#### Tools available for the clinical examination

When asked about tools available for the clinical neuro-otological examination, almost all participating PCPs indicated that a vibration tuning fork (95%), an otoscope (99%), an eye chart were available and were in use in the majority of offices (70%). In contrast, Frenzel's goggles (39%) and hearing tests including smartphone-based applications (32%) were only available in a minority of PCPs' offices.

#### Most frequent diagnoses made by PCPs in patients presenting with vertigo or dizziness

BPPV was the most frequent diagnosis made by PCPs, ranking first in 79.5% of participants (median and interquartile range [IQR, 25–75%]: 1.0 [1.0; 1.0]). The second most frequently made diagnosis was multifactorial dizziness (being first in 11.9% and second in 21.2% of PCPs, with a median ranking of 3.0 [2.0; 5.0]), whereas a diagnosis of gait imbalance/dizziness related to peripheral polyneuropathy followed in third place (ranking first in 1.3% of PCPs, second in 15.9%, and third in 17.2%, with a median ranking of 4.0 [3.0; 6.0]) (see Table 2 for details).

**Table 2:** Most frequently made diagnoses when assessing the dizzy patient in private practice.

**Diagnoses made (in order of decreasing frequency)**	**Ranking (median, interquartile range [25%; 75%])**
Benign paroxysmal positional vertigo (BPPV)	1.0 [1.0; 1.0]
Multifactorial dizziness	3.0 [2.0; 5.0]
Dizziness/gait imbalance linked to peripheral polyneuropathy	4.0 [3.0; 6.0]
Acute unilateral vestibulopathy	5.0 [3.0; 6.0]
Vertigo or dizziness of unclear origin	5.0 [3.0; 7.0]
Functional dizziness	6.0 [3.0; 7.0]
Vertigo or dizziness related to cardiovascular disease	6.0 [4.0; 9.0]
Menière's disease	7.0 [5.0; 8.0]
Vestibular migraine	7.0 [6.0; 8.0]
**Specialists considered for further evaluation of the dizzy patient (in order of decreasing frequency)**	
Ear-nose-throat (ENT) specialists	1.0 [1.0; 2.0]
Neurologists	2.0 [1.0; 2.0]
Emergency physicians	3.0 [3.0; 4.0]
Interdisciplinary center for assessing vertigo//balance disorders	4.0 [3.0; 5.0]
Cardiologists	5.0 [4.0; 5.0]
Psychiatrists	6.0 [6.0; 7.0]
Neurosurgeons	7.0 [6.0; 7.0]
Spinal cord surgeons	8.0 [7.0; 8.0]
**Lack of specific diagnosis**	
In patients with acute dizziness	
After first consultation	35% [25%; 50%]
Upon completion of the diagnostic workup	20% [10.0%; 34.5%]
In patients with episodic/chronic dizziness	
After first consultation	50% [40%; 65.8%]
Upon completion of the diagnostic workup	31.5% [19.3%; 50%]

PCPs indicated that in a substantial fraction of cases, no specific diagnosis could be reached after the first consultation in both patients with acute dizziness and episodic/chronic dizziness, with more than half of these patients still lacking a specific diagnosis after workup (see Table 2).

#### Diagnosing and treating BPPV

Although almost all PCPs were familiar with the provocation maneuver for testing for posterior-canal BPPV (i.e., the Hallpike-Dix maneuver, 91%), a minority of PCPs were aware of provocation maneuvers for diagnosing lateral-canal BPPV [supine-roll maneuver (32%) and bow and lean test (9%)], as shown in Table 3. Asked about repositioning maneuvers, 93% of PCPs preferred a single repositioning maneuver, with the Epley maneuver (84% for posterior-canal BPPV treatment) being more frequently applied than the Semont maneuver (47%). Only 19% of PCPs indicated being familiar with at least one treatment maneuver for lateral-canal BPPV, with numbers for the Barbecue maneuver (17%) being higher than for the Gufoni maneuver (5%).

**Table 3:** Treatment strategies in dizzy patients.

**Treatment options considered in patients with acute dizziness/vertigo**	**Fractions (%, median [IQR])**
Physical therapy	20% [5.5%; 34%]
Antiemetic drugs	46% [20%; 70%]
Anti-vertiginous drugs	50% [20%; 75%]
**Treatment options considered in patients with episodic or chronic dizziness/vertigo**	**Fractions (%, median [IQR])**
Physical therapy	40% [20%; 69%]
Antiemetic drugs	12% [5%; 30%]
Anti-vertiginous drugs	40% [20%; 60%]
Betahistine	144/152 (95%)
Ginkgo biloba extract	98/152 (64%)
Cinnarizine + Dimenhydrinate	68/152 (45%)
Flunarizine	34/152 (22%)
Steroids	30/152 (20%)
**Diagnostic and therapeutic procedures in patients with (suspected) BPPV**	
Diagnostic maneuvers in BPPV known and applied by PCPs	Fractions (%)
Hallpike–Dix maneuver	138/152 (91%)/137/152 (90%)
Supine roll maneuver (90° barbecue maneuver)	48/152 (32%)/38/152 (25%)
Inverse Hallpike maneuver (for testing for anterior canal BPPV)	32/152 (21%)/25/152 (16%)
Bow and lean test	14/152 (9%)/11/152 (7%)
Therapeutic maneuvers in BPPV performed by PCPs	
Epley maneuver	128/152 (84%)
Semont maneuver	72/152 (47%)
Gufoni maneuver	8/152 (5%)
Barbecue maneuver	26/152 (17%)
Others	1/152 (1%)

BPPV, benign paroxysmal positional vertigo; IQR, interquartile range.

A large majority of PCPs recommended home exercises for self-treatment of BPPV, providing verbal instructions (70/24%; always/often) or brochures/drawings (48/26%). Referring to web-based teaching videos for self-repositioning maneuvers was always/often true for 23/30% of participating PCPs. About half of PCPs indicated that they always or often prescribed anti-vertiginous drugs (12/34%) or antiemetic drugs (5/47%) to patients with suspected BPPV. Few PCPs indicated that they always (3%) or often (6%) prescribed vitamin D to patients with recurrent BPPV.

#### Referral patterns of PCPs for dizzy patients and triggers for further evaluation

**Figure 4:**
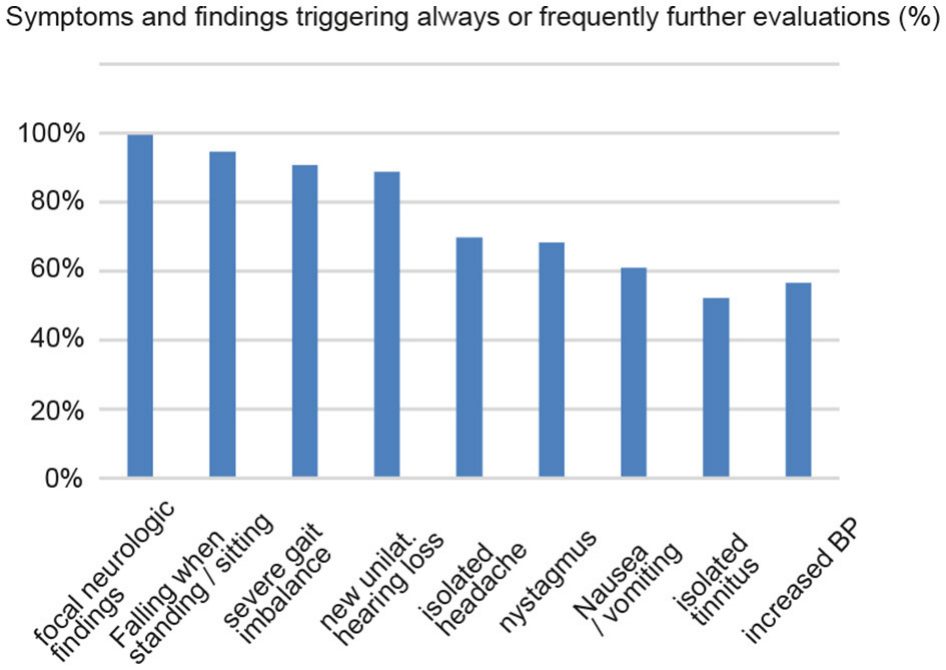
PCPs indicated in what percentage of cases various symptoms and findings will always or frequently trigger further evaluation.

PCPs indicated that approximately one-third (30% [20; 50]; median [IQR]) of all acutely dizzy patients were referred to specialists, whereas every second patient (50% [20.3; 75]) with episodic or chronic dizziness was sent to a specialist for further evaluation. Specialists most frequently considered were ENT specialists (ranking: 1.0 [1.0; 2.0]), neurologists (ranking: 2.0 [1.0; 2.0]), and emergency physicians (ranking: 3.0 [3.0; 4.0]) (see Table 2 for details). In patients with acute dizziness, participating PCPs agreed that the presence of various symptoms or findings will always or frequently trigger further evaluation with high rates, as illustrated in Figure 4.

When performing a univariable regression analysis with regard to the odds of referring an acutely dizzy patient to a specialist, the location of the PCP's office, the number of physicians working in the PCP's office, the number of dizzy patients seen per month, and an unclear diagnosis after the PCP's initial assessment showed significant effects. Specifically, PCPs with an office in the Latin part of Switzerland (OR = 0.66 [0.48–0.91], *p* = 0.011) and those PCPs that saw fewer dizzy patients per month (OR = 0.98 [0.97–0.99], *p* = 0.004) made significantly fewer referrals to specialists, whereas significantly more referrals were made by PCPs working in large offices (five or more physicians) compared with offices with a single physician (OR = 1.65 [1.09–2.50], *p* = 0.019) and with increasing fractions of acutely dizzy patients receiving no specific diagnosis (OR = 1.12 [1.01–1.23], *p* = 0.025). This was confirmed in a multivariable analysis (see Supplementary Table S1).

### Treatments prescribed for dizzy patients by PCPs

Among a selection of treatment options for dizzy patients proposed, a minority of PCPs selected prescribing physical therapy (20%) or antiemetic drugs (46%) in acutely dizzy patients, whereas half of the participating PCPs agreed to the use of anti-vertiginous drugs in these patients (50%). In patients with episodic or chronic dizziness/vertigo, rates for prescribing physical therapy were higher (40%), whereas anti-vertiginous drugs and antiemetic drugs were prescribed less frequently than in acutely dizzy patients (see Table 3 for details). Among the anti-vertiginous drugs given, most often betahistine and ginkgo biloba extracts were recommended.

### Actions taken in the patient with suspected acute unilateral vestibulopathy

A majority of participating PCPs indicated that it was always (34%) or often true (46%) that patients with a diagnosis of (suspected) acute unilateral vestibulopathy are sent for further evaluation/treatment to an ENT specialist or a neurologist. In contrast, a smaller fraction of PCPs indicated that they would refer such patients always (15%) or often (40%) to an emergency physician. A minority of PCPs agreed that they would always or often order a computed tomography (CT) scan (always: 3%, often: 9%) or magnetic resonance imaging (MRI) (always: 8%, often: 27%) in patients with (suspected) acute unilateral vestibulopathy.

When performing a univariable regression analysis with regard to the odds of ordering a brain MRI in patients with suspected acute unilateral vestibulopathy, the number of years of professional experience (*p* = 0.059) and the location of the PCPs' office (*p* = 0.078) showed no significant impact (see Supplementary Table S2).

With regard to treatment strategies in patients with acute unilateral vestibulopathy, a majority of PCPs indicated that they would initiate symptomatic treatment with antiemetics (25/54%; always/often true) or anti-vertiginous drugs (24/32%). Prescribing steroids (21/27%) or antiviral drugs (3/12%) was considered by a minority of participating PCPs only.

For patients with acute vertigo or dizziness, there were no significant correlations between the number of dizzy patients seen per month by the PCPs or the fraction of patients receiving no specific diagnosis after initial assessment and the frequency of prescribing physical therapy, antiemetics, or anti-vertiginous drugs (see Supplementary Table S3).

### Actions taken in the patient with episodic/chronic vertigo or dizziness

A majority of participating PCPs indicated that it was always (31%) or often (55%) true that patients with episodic/chronic dizziness/vertigo are sent for further evaluation/treatment to an ENT specialist or a neurologist. In contrast, a smaller fraction of PCPs indicated that they would refer such patients always (14%) or often (45%) to an interdisciplinary vertigo/balance center. When performing a univariable regression analysis with regard to the odds of referring patients with episodic or chronic dizziness/vertigo to a specialist, those PCPs aged 30–40 years demonstrated significantly increased odds (2.20 [1.01–4.81], *p* = 0.048) compared with those PCPs aged more than 60 years. Furthermore, referrals to specialists were significantly correlated with the fraction of dizzy patients (episodic or chronic) receiving no specific diagnosis after the initial assessment (OR = 1.18 [1.08–1.30], *p* < 0.001). This was confirmed in a multivariable analysis (see Supplementary Table S4).

Only a minority of participating PCPs indicated that they would always (14%) or often (27%) perform provocation maneuvers for possible BPPV in patients presenting with episodic or chronic dizziness/vertigo. With regard to treatment strategies in patients with episodic or chronic vertigo/dizziness, a majority of PCPs indicated that they would prescribe physical therapy for balance training (23/55%; always true/often true) and initiate symptomatic treatment with anti-vertiginous drugs (11/53%), whereas a minority would prescribe antiemetic drugs (4/32%). Only a few PCPs indicated that they would take no action (2/8%; always/often true) but only follow up on these patients. In patients presenting with episodic/chronic dizziness or vertigo, the odds of prescribing anti-vertiginous drugs were significantly increased (OR = 1.01 [1.00–1.03], *p* = 0.034) for those PCPs that see larger numbers of dizzy patients per month. Notably, no significant correlations were observed for other treatments (physical therapy and antiemetics) and the number of dizzy patients seen and for any treatment when put into relation with the fraction of patients receiving no specific diagnosis after the initial assessment (see Supplementary Table S3).

## Discussion

This online survey was driven by three distinct aims. Specifically, it was designed to (a) gain more knowledge about the current exposure of PCPs to dizzy patients, (b) identify limitations and pitfalls in the diagnostic workup, and (c) ask for the specific needs of the PCPs. In this publication, we focus on the first two aims, whereas the third aim will be addressed in a companion article. Gaining more knowledge about the current state of care for the dizzy patient in Switzerland from the PCPs' perspective is an important prerequisite to understanding current limitations and needs.

Overall, most participating PCPs worked at offices located in cities or agglomerations, reported long-standing professional experience, and spent more time with a dizzy patient than with an average patient. While they reported being familiar with most aspects of history taking in the dizzy patient, considerable differences in the role of applied bedside examination techniques were found (see Table 4 for a summary). While taking a general neurological examination and testing for BPPV or looking for spontaneous nystagmus with fixation preserved was done by most, other exams were less often performed [including the HINTS exam (20)]. In general, in patients presenting with episodic or chronic vertigo/dizziness, a specific diagnosis was reached less often than in acutely dizzy patients. Treatment strategies depended strongly on the suspected cause of dizziness, with antiemetics, anti-vertiginous drugs, and physical therapy being the most popular.

**Table 4:** Key findings from the questionnaire.

	**Indicated by (%)**	**Familiarity/rate of agreement**
**History taking**		
Strong agreement that asking about body movements that triggered dizzy spells is important	95%	Very high
Strong agreement that asking about the type of dizziness is important	92%	Very high
Strong agreement that asking about accompanying symptoms is important	79%	High
Strong agreement that asking about current medication is important	66%	Moderate
Strong agreement that asking about recent head or neck trauma is important	59%	Moderate
**Bedside exam**		
Performing provocation//repositioning maneuvers when posterior-canal BPPV	90%/93%	Very high
Performing provocation//repositioning maneuvers for lateral-canal BPPV	25%/19%	Low
Strong agreement that looking for spontaneous nystagmus with fixation preserved is important	77%	High
Strong agreement that performing a general neurological examination is important	73%	High
Strong agreement that performing the head-impulse test is important	47%	Low
Strong agreement that looking for spontaneous nystagmus with fixation removed is important	42%	Low
Strong agreement that performing the alternating cover test is important	21%	Very low
**Diagnostic workup/referrals**	**Fraction (%, median [interquartile range])**	
No specific diagnosis reached		
Acutely dizzy patients	35% [25%; 50%]	
Patients with episodic/chronic vertigo or dizziness	50% [40%; 65.8%]	
Referral to specialists		
Acutely dizzy patients	30% [20; 50]	
Patients with episodic/chronic vertigo or dizziness	50% [20.3; 75]	

BPPV, benign paroxysmal positional vertigo.

### Epidemiological aspects, history taking, and clinical examination

A total of 152 completed surveys were returned, with fractions proportional to the different language areas in Switzerland. However, the cohort was skewed toward male PCPs (74%) located in agglomerations or cities (81%) with long-standing working experience (26.1 ± 8.9 years, average ±1 SD). Thus, other groups including female PCPs and less experienced PCPs were under-represented, potentially limiting the generalizability of the reported findings.

We found that PCPs are very familiar with the concept of dizzy spells triggered by certain body movements (full approval: 95%), whereas asking for situational triggers (looking especially for functional dizziness) is considered less important (full approval: 69%). Likewise, almost all participating PCPs agreed for sure that receiving a description of the type of dizziness is important (92%), whereas rates for asking for the frequency of attacks (full approval: 72%) and the duration of single attacks (full approval: 70%) were lower. This is against modern concepts of addressing patients' complaints as proposed by Newman-Toker and Edlow [TiTrATE approach, (23)]. Overreliance on the type of dizziness has been shown to be dangerous, as patients are inconsistent in describing their sensations and physicians have distinct concepts in interpreting reported dizzy complaints. Thus, when relying on the type of dizziness, certain diagnoses may be favored or discarded, increasing the risk of misdiagnosis and delayed or even missed treatment (24).

With regard to bedside examination techniques, PCPs are well aware of the importance of performing provocation maneuvers in patients with suspected BPPV (very important: 84%) and of the importance of looking for spontaneous nystagmus (with fixation preserved) in dizzy patients (very important: 77%), whereas performing a general neurological examination (very important: 73%), an analysis of stance (very important: 68%) and gait (very important: 59%), and looking for spontaneous nystagmus with fixation removed (very important: 42%) were considered somewhat less important, with the latter one being well-explained by the finding that only 39% of participating PCPs had Frenzel goggles available. More subtle oculomotor or vestibular tests were considered very important only in about half of the PCPs or less, with head-impulse testing (very important: 47%) and looking for a vertical skew (very important: 21%) having the lowest rates. Likewise, assessment of hearing was considered very important in only 38% of cases (finger rubbing). Thus, knowledge about key elements linked to high diagnostic accuracy for central causes in acutely dizzy patients (HINTS paradigm) was limited among PCPs. We hypothesized that such bedside diagnostic tools might be more popular with younger PCPs. In our survey, however, we did not find any effect of age on the use of HINTS(+) in a multivariate regression analysis. This potentially indicates that continuous education provided to PCPs of all ages likely does not cover modern concepts of how to diagnose the acutely dizzy patient.

### Referral patterns

In the case of an (suspected) acute unilateral vestibulopathy, this usually triggers a referral to a neurologist or an ENT specialist (indicated by 80% of PCPs), whereas referrals to an emergency department were considered less frequent (being always or often the case in 45% of participating PCPs only). This referral pattern indicates a perceived overall low to intermediate urgency for these patients for further evaluation only. Regression analyses identified several parameters that significantly affected the referral pattern, including the location of the PCPs' office {with lower odds of referral for those located in the Latin part of Switzerland (OR = 0.66 [0.48–0.91], *p* = 0.011)} and the number of dizzy patients seen per month {with slightly lower odds for those PCPs that saw fewer dizzy patients per month (OR = 0.98 [0.97–0.99], *p* = 0.004)}. We can only speculate about the reasons for such regional differences in the referral pattern of acutely dizzy patients. Potentially, they arise from differences in aiming for diagnostic confirmation, patients' preference for further diagnostic workup, judgment of urgency, or low-threshold accessibility to nearby specialists.

In contrast, increased odds ratios were found for PCPs working in large offices (five or more physicians; OR = 1.65 [1.09–2.50], *p* = 0.019) and for those with higher fractions of acutely dizzy patients receiving no specific diagnosis (OR = 1.12 [1.01–1.23], *p* = 0.025). While the latter pattern can well be explained by expanding the diagnostic workup in the case of an unclear presentation, the first pattern (more referrals from larger PCP offices) was unexpected. We initially hypothesized that being able to discuss such cases with colleagues would lower the odds of referrals. Possibly, such discussions rather facilitated referral, especially if such larger offices were interdisciplinary and the required specialist was available in-house.

### Imaging in acutely dizzy patients

Current guidelines on the management of the acutely dizzy patient, such as the GRACE-3 consensus statement (25), do not recommend the use of CT-based imaging unless the patient has a suspicion of bleeding, inner ear fistula, or dissection. In our survey, only a minority of PCPs indicated that they always or often order brain imaging (CT 12%, MRI 34%) in acutely dizzy patients. Notably, the decision not to order brain imaging in the majority of cases and asking for non-urgent specialized assessment emphasizes the PCPs' confidentiality with the clinical diagnosis made. According to the GRACE-3 consensus statement, MRI-based imaging should not be used as a first-line test if a clinician trained in HINTS is available (25). However, in our survey, only a minority of participating PCPs identified the key bedside tests for reliably differentiating between peripheral and central causes in acute prolonged vertigo and dizziness [i.e., HINTS(+) (22)] as being very important, which is also reflected in the distribution of the HINTS(+) score in Figures 4B, C. Furthermore, early MR imaging bears the risk of false-negative findings (26). This is especially true for small brainstem strokes (27).

### Treatment strategies in acutely dizzy patients

Notably, in the context of (suspected) acute unilateral vestibulopathy, symptomatic treatments (i.e., antiemetics and anti-vertiginous drugs) were more popular than the application of steroids. Specifically, only 46% of PCPs indicated that they often or even always prescribe steroids for this condition. This probably reflects the ongoing discussion on the value of steroid treatment in acute unilateral vestibulopathy with diverging recommendations (28, 29). To what extent accompanying diseases (such as diabetes or psychiatric co-morbidities) have biased this decision has not been addressed in this questionnaire. With a majority of PCPs often or always prescribing antiemetics (79%) or anti-vertiginous drugs (56%) in the setting of acute unilateral vestibulopathy, this needs further discussion. Whereas vestibular suppressants may be utilized in the acute stage, they may inhibit central vestibular compensation when taken for more than 2–3 days and are thus largely inappropriate (30). Notably, we did not assess the duration of the planned prescription of vestibular suppressants in our questionnaire, limiting our conclusions based on this observation. Considering non-pharmaceutical treatment strategies in acutely dizzy patients in general, only a minority (20%) of PCPs indicated prescribing physical therapy in this setting, which is in line with previous reports proposing that clinical providers (including PCPs, neurologists, ENT specialists, and audiologists) are frequently unaware of the concept of vestibular rehabilitation (31). Limiting treatment costs may be an alternative explanation for why referral rates for physical therapy by clinical providers are low. Based on the referral patterns indicated by the PCPs participating in our survey, 4 out of 5 patients will not receive physical therapy, despite its proven value (19). This potentially prolongs recovery and may also negatively affect the outcome of acutely dizzy patients.

### Referral patterns and treatment strategies in patients with episodic or chronic dizziness

While BPPV is considered the most frequent cause of episodic vertigo or dizziness worldwide (32), only 41% of participating PCPs performed testing for BPPV in patients with episodic or chronic dizziness on a regular basis (i.e., often or always), despite being aware of the diagnostic/therapeutic maneuvers for posterior-canal BPPV as indicated by >90% of participants. While a previous study reviewing medical records of dizzy patients found that 89% of providers (consisting of PCPs, ED physicians, and various specialists) did not evaluate a patient for BPPV by examining for positional nystagmus (18), and in another study only 3.9% of acutely dizzy patients presenting to the ED received a Dix–Hallpike test (33), the numbers identified in our survey (indicating the PCPs' intention to perform such testing) are higher but still indicate that screening for BPPV is not consistently performed.

At the same time, 86% of participating PCPs indicated frequent referral of patients with episodic or chronic dizziness/vertigo to neurologists or ENT specialists for further evaluation, whereas referral to interdisciplinary dizziness clinics was considered (often or always) less often (59%). Thereby, the odds of referring patients with episodic or chronic dizziness/vertigo to a specialist were significantly higher for PCPs aged 30–40 years compared with those aged 60 years or more (2.20 [1.01–4.81], *p* = 0.048). This referral pattern could either be interpreted by more limited professional experience or by a more interdisciplinary approach to the dizzy patient perceived by younger PCPs. This could be supported by the observation that significant shifts in diagnoses in dizzy patients can be observed when referring to specialists. Specifically, after a diagnostic work-up in an academic tertiary dizziness center, the fraction of patients diagnosed with “unclear dizziness” decreased from 70 to 10%, mainly due to a near doubling of the patients diagnosed with BPPV (34). Likewise, a change in diagnosis in 67% of patients after assessment by specialized neuro-otologists was reported in another study (35). Not surprisingly, referrals to specialists were significantly correlated with the fraction of dizzy patients receiving no specific diagnosis after the PCP's initial assessment in our study (OR = 1.18 [1.08–1.30], *p* < 0.001). Importantly, dizzy patients may also consult physicians of different specialties simultaneously (36), resulting in unnecessary or redundant medical examinations, causing a financial burden to the healthcare system, and increasing waiting times for specialists' assessments (2, 37).

Thus, with a referral rate of >80%, this underlines the need for improvement in the diagnostic workup of these patients at the PCP's office, potentially reducing the number of referrals to specialists. This includes screening more consistently for BPPV by performing provocation maneuvers in patients with episodic/chronic vertigo or dizziness, but also for functional dizziness by asking about specific situations/locations that may trigger dizzy spells (which was indicated to be important only by 68% of PCPs). Lack of time during the PCPs' busy schedule (spending 20.7 ± 5.7 min per patient on average and requiring more time in the majority of dizzy patients), however, will be one major challenge when implementing such recommendations.

Notably, three-quarters of PCPs indicated the frequent prescription of physical therapy in patients with episodic or chronic dizziness, emphasizing the popularity of non-pharmaceutical treatment strategies in this setting. When asked whether or not to prescribe physical therapy in patients with episodic or chronic dizziness/vertigo, however, only 40% of PCPs indicated to do so, indicating a certain discrepancy compared to when asked about the likelihood of prescribing physical therapy in this setting.

The high referral rates observed in our survey are in contrast to significantly lower rates previously published in the range of 14.9% (15), 16% (38), 22% (18), and 47.8% (39). These discrepancies could be related to differences in the study design [comparing results from a prospective survey assessing the PCPs' intentions with retrospective chart reviews reporting on ordered referrals (15)], distinct national healthcare systems, including differences in access to specialists, coverage of costs of referrals, and waiting time for referrals (reducing the probability of a referral in a healthcare system with long waiting times), or patient populations studied.

### Diagnosing and treating benign paroxysmal positional vertigo

In this survey, we found a surprising difference in PCPs' knowledge about diagnosing and treating BPPV depending on the canal affected. While a vast majority of PCPs indicated being very familiar with performing diagnostic (90%) and therapeutic (93%) maneuvers for posterior canal BPPV, rates for diagnostic (25%) and therapeutic (19%) procedures performed for suspected lateral canal BPPV were significantly lower. Overall, the Epley maneuver was applied more frequently than the Semont maneuver (84 vs. 47%), and the Barbecue maneuver was more popular than the Gufoni maneuver (17 vs. 5%). Taking into account that both repositioning maneuvers for posterior canal BPPV are considered equally effective (40), regular use of either repositioning maneuver is sufficient. However, with a rate of approximately 5–15% of all BPPV patients suffering from non-posterior canal (mostly lateral canal) BPPV (32, 41), and as repositioning maneuvers for lateral-canal BPPV are feasible in the PCP's office as well, this indicates a gap in knowledge that should be addressed. Likewise, only 20% of PCPs fully agreed to being well trained for diagnosing and treating BPPV, despite BPPV being found to be the most frequently made diagnosis by participating PCPs, further emphasizing the need for improved diagnostic skills (42) and teaching activities focusing on (lateral) canal BPPV.

Furthermore, about half of PCPs often or always prescribe anti-vertiginous drugs or antiemetic drugs to patients with suspected BPPV, which is against best practice (32, 43) and consistent with an earlier study reporting medical treatment with antiemetics, antihistamines, or anti-inflammatory medication for more than 50% of the patients (37).

### Study limitations

This study has several limitations that need to be considered. First, participation in this online survey was optional, and thus a selection bias (e.g., based on the PCP's interest in taking care of the dizzy patient or the current workload not allowing them to spend time to fill out a survey) cannot be excluded. With more than 8,500 registered PCPs working in Switzerland and an invitation for participation in our survey sent to 5,668 PCPs, our sample of 152 completed questionnaires represents only a small fraction of all PCPs contacted (2.68%). Potentially, the respondents to our survey had a higher chance of having an interest in managing dizzy patients, which would overestimate the current practice and knowledge on neuro-otology among PCPs working in Switzerland. Thus, neurotology practice in the real world may be less optimal than reported in our survey. Second, we collected data on the PCPs' self-reported diagnostic and therapeutic procedures, which may diverge from the actually executed procedure in a specific patient. Third, with regard to the reported numbers on the exposure to dizzy patients in daily practice, there is a risk of recall bias (with over- or under-estimating actual numbers and diagnoses made). Fourth, we did not collect any information about the participating PCPs' curriculum, which might be very variable and may include ear-nose-throat and/or neurology training in some PCPs.

## Conclusion

Participating PCPs reported being familiar with most aspects of history taking in the dizzy patient; however, only a minority of PCPs were familiar with HINTS bedside testing and with lateral-canal BPPV treatment and had Frenzel's goggles available. In general, in patients presenting with episodic or chronic vertigo/dizziness, a specific diagnosis was reached less often than in acutely dizzy patients. The overall referral rates to specialists were substantially higher (>80%) than previously reported, potentially being related to a more permissive Swiss healthcare system. Furthermore, only 41% of PCPs indicated regular screening for suspected BPPV, and a majority of PCPs used vestibular suppressants against current evidence. Such gaps in the PCPs' knowledge in the diagnostic workup and treatment of the dizzy patient need to be addressed, which is discussed in detail in the companion article (44).

## Data availability statement

The original contributions presented in the study are included in the article/Supplementary material, further inquiries can be directed to the corresponding author.

## Author contributions

AZ: Conceptualization, Methodology, Validation, Writing—review and editing. GM: Conceptualization, Methodology, Validation, Writing—review and editing. DH: Data curation, Formal analysis, Validation, Writing—review and editing. HK: Methodology, Writing—review and editing. SD: Methodology, Writing—review and editing. RK: Methodology, Writing—review and editing. AK: Methodology, Writing—review and editing. CC: Methodology, Writing—review and editing. AW-L: Methodology, Writing—review and editing. AT: Writing—original draft, Writing—review and editing, Conceptualization, Formal analysis, Funding acquisition, Methodology, Supervision, Validation.
